# Managing severe malaria in an intensive care unit contraindications and complications of treatment with quinine

**DOI:** 10.1186/2197-425X-3-S1-A350

**Published:** 2015-10-01

**Authors:** M Neno, T Marques, SE Paulo, CM Santos

**Affiliations:** Hospital de Santa Maria, Serviço de Doenças Infecciosas, Lisboa, Portugal

## Introduction

Although Portugal was one of the first countries eradicating malaria in Europe, the burden of the disease remains high, particularly due to the close relationship with its former african colonies. Due to its adverse effects and higher mortality rate in patients treated with quinine, methanalysis by the Cochrane Group published in June 2012, suggests artesunate as first line therapy in both children and adults with severe malaria, particularly cerebral malaria.

## Objectives

To investigate the clinical impact as well as the efficacy and adverse effects of quinine based regimes in the treatment of malaria.

## Methods

We did a retrospective, observational study reviewing the files of all admitted patients between 2009 and 2015 in our specialised Infectious Diseases ICU. We selected all cases with confirmed infections by *P. falciparum*, and registered parameters like age, gender, nationality, country of transmission, severe malaria criteria, indications and contraindications of intravenous quinine, as well as its adverse effects and clinical outcome.

## Results

A total of 24 patients were included in the study, 18 (75%) males, all adults with an average age of 41,3 ± 13,3 years. We consider that 15 (62,5%) patients had developed partial immunity as considered in persons living in an endemic area for more than six months. All cases were imported from endemic countries in Africa. No patients underwent prophylaxis for malaria. The mean time between onset of the symptoms and hospital admission was 4,3 ± 2,2 days. Parasitemia, quantified in all but 2 patients, was, on average, 18,5% ± 15,2 (from 2 to 60%), wherein 18 (81,8%) patients developed parasitemia greater than 5%. Severe malaria, as defined by the WHO, was observed in 22 (91,7%) of the patients, 15 of whom (68,2%) had 4 or more criteria. The mean Apache II score at admission was 16,8 ± 7,7 (from 1 to 33) and the SOFA score was 11,4 ± 6,1 (from 4 to 24), either without correlation with the observed intra-hospitalar mortality rate (8,3%; n = 2). Overall, 20 (83,3%) of the patients were treated with intravenous quinine, being the loading dose between 750 and 1750mg. The mean time between the onset of therapeutic and parasitemia < 1% was 2,3 ± 1,2 days (from 0,25 to 4). The most common adverse effects of quinine, besides the expectable cinchonism, was mild hypoglycaemia (50%; n = 12). Three (12,5%) patients needed aminergic support after initiating quinine. Also 3 patients (12,5%) developed non-severe arrhythmia.

## Conclusions

Although the study is small in size, it confirms the efficacy and reasonable tolerability of quinine based regimes, as opposite to what is described with quinidine that is more cardiotoxic. As so, the true role of artesunate in an experienced and well equipped intensive care unit remains obscure. Attending to the risk of late haemolytic anaemia, its use should be subject of further benefit/risk studies especially in cases of cerebral malaria and for extremely high parasitemia.

